# Voices of unpaid carers: problems and prospects in accessing palliative care and self-care information, resources and services

**DOI:** 10.1177/26323524241255386

**Published:** 2024-05-29

**Authors:** Kristine Van Dinther, Sara Javanparast

**Affiliations:** Research Centre for Palliative Care, Death and Dying, Flinders University, Bedford Park, GPO Box 2100, Adelaide, SA 5001, Australia; Research Centre for Palliative Care, Death and Dying, Flinders University, Adelaide, SA, Australia

**Keywords:** knowledge, palliative care, resources, self-care, support, unpaid carers

## Abstract

**Background:**

Unpaid carers make a substantial contribution to the health economy and carers of palliative patients are particularly vulnerable due to special patient needs and excessive carer burden. The Australian Government recently implemented the Integrated Carer Support Service Model to provide a range of free services to carers in the community. However, it is unclear whether such initiatives are effective and, more importantly, how carers of palliative patients gain access to information, support and services for the patient and themselves.

**Objectives:**

We sought to investigate unpaid carers’ experiences in accessing information and resources for support with patient care with a specific focus on palliative care resources and to determine carers’ access to information and support for self-care. We also aimed to identify what opportunities and challenges remain for these particular carers according to their experiences.

**Methods:**

We conducted 18 semi-structured interviews and 3 focus groups with unpaid family or friend carers of palliative patients in South Australia from metropolitan, regional and rural communities. Grounded in a descriptive phenomenological paradigm, we conducted a hybrid approach to thematic analysis combining deductive and inductive coding following Fereday and Muir-Cochrane’s method.

**Results:**

The government’s web-based initiative provided little impact in supporting carers from our cohort. There remains a substantial gap between the formal recognition of the importance of carers and their lived reality. This recognition by health professionals is vital, as carer self-identification is not common and affects help-seeking behaviour. Carers seek and respond to more grassroots, personalized forms of support and sharing of information.

**Conclusion:**

The lack of self-identification affects carers’ help-seeking behaviours. Carer identification and recognition need to be initiated by health professionals in a proactive manner to ensure carers are prepared for their role and are emotionally supported to sustain it. Carers seek face-to-face guidance and sources of information.

## Introduction

According to the Australian Bureau of Statistics, in 2021, 2,476,681 people provided unpaid assistance to others with a disability, long-term health condition or due to old age. This represents 11.9% of the population over the age of 15 years and almost a percentage point increase on data from 2016.^1^ Census data from the Office of National Statistics in the United Kingdom shows similar trends of the prevalence of unpaid carers. In 2021, Wales and England reported figures of 10.5% and 8.9% respectively.^2^ In the United States, the percentage of unpaid carers is much higher, at around 21%.^3^ It is clear that the role of family and friends caring in the community is becoming increasingly essential to the quality of life of many and an integral support for our health system. This is why carers and meeting their needs have become a major focus for governments, employers, policymakers, practitioners and researchers.^4^

Australia’s ageing population will continue to place pressure on the provision of informal care, the latest projection being that by 2057, 22% of the population will be over 65 years of age.^5^ With our ageing population comes an inevitable increase in demand for palliative care. We define palliative care as patient and family-centred care for people who have a life-limiting illness where the health focus is not on cure but quality of life.^6^ We know that demand for this health care will double in the next 10 years, making it doubtful that primary health professionals will be able to meet this growing demand.^7^ Although unpaid carers of palliative patients provide valuable support and positive outcomes for patients, they also make a significant contribution by providing much-needed support to our health systems.^7,8^ If we calculated the total hours spent on informal care (all types) provided in Australia by unpaid carers in 2020, the replacement cost of these hours with formal care would amount to $77.9 billion dollars.^5^ Even though this figure includes all categories of people requiring assistance and care, it serves to demonstrate the staggering economic savings they permit our health systems. In the United Kingdom for example, data from 2021 showed that unpaid carers contribute 162 billion pounds a year, exceeding the National Health Service (NHS) spending budget of 156 billion pounds for 2020/2021.^9^ In the United States, the estimated value of unpaid carers contributions also vastly exceeds the value of paid home care in the health economy contributing a staggering $470 billion dollars.^10^ Henceforth, unpaid carers in the home have become increasingly recognized as playing an essential role in patient care and are a vital part of the care team.^11,12^ However, although formally acknowledging their role as integral to patient support and recognizing that their numbers are rising, evidence suggests that unpaid carer recognition and health are either ignored or inadequate.^13^

Recently, an Australian study of three different home palliative care service sites showed a lack of structured intervention for carers to assist in preparing them for their role and revealed that only one of the sites assessed carers’ needs.^14^ Other barriers which continue to pose problems for carers are geographical locations^7,14^ and a lack of communication from health care teams regarding the range of provisions and services available to them.^13,15^ One study showed that two-thirds of those with unmet support needs were significantly more likely to identify training and aids as an unmet need to carry out their caring role,^16^ and another study found that lack of communication with health care professionals was the most widely reported unmet need for both patients and carers.^17^ A recent Australian study showed that 39% of carers reported unmet needs in carrying out their role which not only affected patient support but carer well-being.^18^

Unpaid carers’ support in protecting their own health is vital. Therefore, carers require knowledge and information not just regarding resources they can access in the care of the patient but also with regard to resources and support for their own health. Evidence suggests that support for their well-being continues to fall short.^11,19^ Although all carers face challenges, unpaid carers of palliative patients face specific obstacles in their roles and duties, along with experiencing the prospect of facing the death of a loved one. The stresses of caring for a dying friend or family member cannot be underestimated. Physical and mental stresses such as physical exhaustion, emotional distress, social isolation and financial burdens face greater risks to their mental health.^20,21^ As a group, they also face specific psychosocial and physical burdens not just during the caring process but post-bereavement.^22^ Studies also show that distress and anxiety whilst undertaking the role of carer can also arise from a general unpreparedness for the role due to the specific challenges which come from caring for a patient at the end of life.^19,23^ However, the caveat here is that carers are often so immersed and fatigued by the burdens of caring that they rarely consider their own health needs in the process.^11,13^ This is also evidenced in another study, which showed that the allocation of help for family carers depended mostly on whether family carers clearly expressed the need for support.^19^ Interestingly, studies show that in many cases, knowledge about and the organization of resources and support for carers’ well-being often comes through carer interaction with nursing staff.^15,24^

One avenue though which certain organizations have attempted to improve the information and support needs of unpaid carers is through web-based initiatives and film media. In Ireland, a web-based resource was developed specifically for unpaid carers of palliative patients. As an information resource for caring and self-care, it was developed as a collaborative effort between health professionals and carers to identify the core information needs of this particular group.^25^ In Vietnam, investigation into what content would assist in the development of web-based initiatives to help carers of cancer patients was also carried out in a collaborative way. Although the site is yet to be developed, the initiative’s goal was to focus on relevant content and interface useability.^26^ Even though the success of these programmes in terms of carer support is yet to be proven, their strength lies in consulting carers to determine what information they need for both the patient and themselves. One initiative has been tested in Australia. The Victorian Centre for Palliative Care developed a consumer-led project resulting in a DVD information resource for carers of palliative patients. The feedback on its usefulness as a resource for these carers was predominantly positive.^27^ The trend of consulting the end-user on content and needs in the development of such programmes makes logical sense.

In 2019, the Australian Government implemented the Integrated Carer Support Service Model to provide a range of free in person, online and phone-based support services.^28^ Full roll-out was implemented in April 2020, delivering services through various organizations across Australia known as Carer Gateway service providers. In South Australia, the portal is hosted by Carers SA. It serves as a central platform through which a host of not-for-profit, community and government services can be accessed. All carers can search for information, resources and support in their caring role, such as access to counselling and respite care, form community connections, develop skills and learn about government aid and support. The extent to which these services are utilized, accessible and meet the needs of unpaid carers is uncharted.

Although web-based resources for unpaid carers can be a very effective tool to source information and support, they are not always known to carers or access to them comes too late in the care trajectory.^12^ Indeed, one would hope that engagement with health professionals would be the means through which carers were guided to such support networks and sources of relevant information. One could even say that the success of such initiatives may be contingent on such referral.^25^ Irrespective of this, evidence suggests that many carers continue to experience a lack of practical support in terms of access to information and services they need to carry out their role.^14,16^

Instead of grouping all carers together, we sought to focus on unpaid carers of palliative patients. The purpose of our specific focus is that carers of palliative patients are a particularly vulnerable carer group with greater needs and health risks both during the caring process and post-bereavement.^29,30^Although acknowledging that the government initiative exists to support carers, it was not our intention to measure the efficacy of this initiative, but to determine how and where unpaid carers of palliative patients access information, knowledge and support in their role; and how they find information and support for self-care. We sought to determine their existing challenges and the potential opportunities for solutions. Hence, we contribute new insights into how this particular group can be better supported by being the first to demonstrate the links between carer identification and help-seeking behaviours of carers of palliative patients. We also present new insights into the causes of the challenges these carers continue to face in accessing information and support and outline approaches which may offer opportunities to improve support according to the carers.

## Methods

We gathered and approached the data collection utilizing a descriptive phenomenological paradigm following Husserl’s concept of the life-world as it relates to lived experience.^31^ This was deemed appropriate to understand carers’ experiences as it takes for granted that experiences are always intersubjectively formed.^32^ Under the paradigm, bracketing and reflexivity were an important part of the data collection process and were adopted to ensure rigour and restrict the possibility of our researcher’s pre-existing assumptions compromising the data.^33^ The lead author was not part of the conceptualization of the research and thus began with no prior bias or assumptions. Further, reflexive rigour was ensured with her background in anthropology, where reflexive practices are central to the anthropological method.

Both researchers were female. The lead author (PhD), working contractually as a research scholar, had extensive experience in qualitative research and reflexive interview methods in the fields of public health and anthropology. The second author (MD, PhD), as a Senior Research Fellow, had extensive experience in public health care policy research and programmes research.

In partnership with Carers SA – a carer’s advocacy and support organization in South Australia – the information regarding our research and our call for participants was widely distributed through social media platforms, government and non-government organizations, and general practices. Carer’s SA provides the portal for information and services provided by the government initiative in that state.

The reporting of this study conforms to the consolidated criteria for reporting qualitative research (COREQ)^34^ (See Supplemental File)

### Recruitment

Recruitment was purposive. We restricted participants to those over the age of 18 who cared for someone above the age of 18. We asked for any carers who were currently caring or bereaved where the patient was receiving palliative care. For bereaved, we restricted the time frame to 2 years after the caring role to ensure recall and to limit the distress that may be caused by taking part. Although we stated in the information sheet that we were looking for carers of palliative patients, other carers who met the criteria were also included. Interested parties contacted the research team, and an information sheet and consent form were provided to them. The researchers had no prior association with the participants. Data collection was carried out between August 2022 and October 2023.

In total, we conducted 18 individual semi-structured interviews and 3 focus groups with unpaid family or friend carers of palliative and other patients in South Australia. The lead author conducted the interviews, and both authors held the focus groups. Study participants were from diverse population groups, including Anglo-Australian, Italian and Greek communities, along with a total of seven carers residing in rural and regional areas.

The interview questions and guide were developed to address three areas of the research aims and objectives. These were carers’ access to palliative care information, resources and services, carers’ access to and knowledge of self-care resources and support, and the identification of gaps in service provisions according to the carers. Some interviews were conducted face-to-face in their homes (*n* = 10), online *via* Teams (*n* = 6) or *via* telephone (*n* = 2). The duration of each interview was between 40 and 90 min. No follow-up interviews were conducted, no other parties were present during the interviews and no participants withdrew.

All 18 interviewees were contacted to elicit interest in being part of our focus groups, and 13 agreed to participate. Focus groups were facilitated by both researchers and were audio-recorded for transcription. The first focus group was conducted online and consisted of four participants; the second group was in person on location and consisted of six participants, and the third group consisted of three participants on location, with one of the participants joining the group online *via* Teams. All groups were given a summary of some of our preliminary findings and key themes, and participants were encouraged to provide their point of view regarding the findings. Although the discussions were guided by the findings, participants were encouraged to share their ideas on how support services can be tailored to address the special needs of carers of palliative patients.

### Analysis

After the completion of the interviews, the digital audio recordings were transcribed by an independent service, and all transcriptions were entered into Nvivo v20 qualitative software manufactured by Lumivero. Transcriptions were not returned to the participants. We undertook a hybrid approach to analysis, which involved both deductive and inductive analysis processes as espoused by Fereday and Muir-Cochrane.^35^ A combined approach, where it does not begin with a theoretical proposition, is guided by the framework of the objective, question and literature. It involves the use of descriptive codes generated by both the research question and the literature – being the deductive phase of analysis – and the generation of themes from the data during the inductive analysis.^36^

Beginning with deductive analysis, the primary researcher created nodes in Nvivo v20 under three broad categories in order to capture data to answer the broader study question. These included (1) carers’ access to palliative care information, resources and services; (2) carers’ access to and knowledge of self-care resources and support; and (3) the identification of facilitators and barriers to support from the carer’s experiences. The codes at this stage were strictly descriptive in nature, reflecting the objective aims of the research and were akin to creating a code-book to organize the data. For example, ‘resource knowledge for patient care’ and ‘resource knowledge for self-care’, ‘sources of information and support’, etc. Once this deductive phase was complete, some common trends in the data were emerging. These trends were summarized to form some preliminary findings and tested against our three focus groups for the purposes of triangulation.^37^

Transcripts from the three focus groups were coded in the identical fashion described above and validity of the initial codes and trends were tested against the responses from the groups. After the focus groups, both authors agreed that the data had reached saturation. Once this process was complete, an inductive analysis of all of the transcripts was carried out to identify dominant themes resulting initially in 9, which were reduced to 6. Minor themes, such as ‘time constraints’ were felt to be already widely acknowledged in the literature for unpaid carers and so were not provided prominence. This reduction process was made possible *via* cross-checking by the second author who tested the themes against two transcripts to ensure the reliability of the codes in accordance with the stage two validation method in Fereday and Muir-Cochrane’s approach. The two researchers then met to discuss the themes which were then refined and collapsed into four main themes.

## Results

We were able to obtain a total of 18 participants in this study. In response to an introductory open-ended question at the beginning of interviews, participants provided information and details about their caring roles, including years as a caregiver, patient illness and other contextual data. All were adult carers over the age of 25. Female carers comprised 83% of our participants and there was a higher percentage of bereaved carers (72%) compared to those currently in the caring role. Over 60% of participants provided care on a full-time basis, and cancer was the most common health condition. A summary of socio-demographic data is presented in Table 1:

**Table 1.** Socio-demographic summary.

Profile of participants	
Gender	Female: 15
	Male: 3
Caregiving duration	<1 year: 4
	1–2 years: 5
	2–5 years: 3
	>5 years: 6
Caring role	Full-time: 11
	Part-time: 7
Relationship to the patient	Spouse: 8
	Son/daughter: 7
	Sibling: 1
	Friend: 2
Patient’s illness	Cancer: 11
	Organ failure (heart, kidney, lung): 2
	Mental illness/dementia/Alzheimer’s: 4
	Multiple system atrophy: 1
Caring status at the time of interview	Current carer: 5
	Bereaved: 13
Employment status during caregiving	Working full-time: 7
	Working part-time: 3
	Not working: 8

Our findings will be presented under the two main categories under which our data was sorted. The first relates to carers’ experiences organizing care for the patient, and the second refers to organizing care for themselves. Data from the third category, facilitators and barriers to support, is in answer to the first two topics and, as such, will be incorporated into the corresponding themes.

### Knowledge of and access to information and services: Organizing care for the patient

Our findings show knowing where to gain access to information about palliative care assistance, home nursing help, government assistance and other support services to care for the patient came from websites, brochures and even word of mouth. Fundamentally, there were some difficulties gaining the knowledge and services that carers required to undertake their role, and some reported a lack of cohesion in the dissemination of knowledge from health professionals that would have helped them better support the patient. Hence, not all participants were aware of everything that they could have access to. Sometimes, even if avenues of support or information were identified, the ability to navigate, access and obtain that support in a timely and efficient manner was problematic.

### Theme 1: General Practitioners and nurses as gateways

General Practitioners (GPs) have the potential to be important sources of information regarding palliative care support services for patients and carers. Our data concurs with this and shows that there were mixed reactions regarding GPs as effective gateways regarding palliative care information and provision, with more positive responses provided with carers’ interactions with nurses (Table 2).

**Table 2.** General practitioners and nurses as gateways.

Theme	Description	Exemplar quotes
General practitioners as gateways	Some of our participants were happy with their GPs and found the support and information they needed through them. At the very least, palliative care and other avenues of support for the patient were discussed:	‘Whereas, can I just say, Mom’s GP was amazing. She’s the one who got us into palliative care, she’s the one who would do home visits. And I would ring her and I’d say, “Mom’s not doing too good. What do you reckon?” And she would say, “Let me do a visit on my way home”’. (Carer 3) ‘Yeah. We saw him on the 12th. And Bruce had stopped his radiology on the 5th. So, a week later, we saw Peter and Peter said, “I’ll send some paperwork to palliative care.” And then he said to Bruce, “If you don’t hear anything, come back and see me”’. (Carer 16) ‘Um, that my husband researched all of that because he set it all up. But I’m pretty sure. As soon as we said to my doctor in Adelaide or his doctor in Adelaide, that we’re just going to go down this route. She gave us the team number up here. . .Yeah, there’s a palliative care team up here and they go they liaise with doctors in North Adelaide somewhere’. (Carer 1)
	Not all participants expressed satisfaction with their doctor. Indeed, many discussions regarding palliative care came from specialists such as oncology doctors. A couple of participants shared their disappointment with the level of information and offers of services and advice provided by the patient’s primary doctor:	‘I disagree with you. . .in the sense where we say that GP’s and all that, it’s not their role. If you’ve got a GP that’s been looking after a patient for a really long time, they should be able to go, “You know what? Now’s the time we start thinking about palliative care”’. (Carer 3) ‘And that’s okay but if they have another word for it or another way of saying that, “You’re at this point where you just need a lot more help. We’re not saying that you’re dying but we’re saying that you now have some chronic conditions,” or whatever, “and so these are the things that you could perhaps do. These are the resources that you might be able to tap into, this is the things that you could go to for help,” then that would work. Because then you’d start to get onto those things early. Because it wasn’t the case that they weren’t necessarily there but by the time you’d found out about them and spent a couple of months to get some approval, and then some of that was six months, then. . .it was too late. . .We got all our stuff too late’. (Carers 11 & 12)
Nurses as gateways	For the most part, participants were positive regarding the information and support provided by the nursing staff and felt they were a good avenue through which more information could be sourced:	‘And one day she said to me when I rang the nurse and I said, “Mum’s not breathing too good.” And I take videos so they could hear her, and she said to me, “I think it’s time we talk about palliative.” And I went, “No, are you telling me my mum’s dying?” And she goes, “No, no, no, no, no. It’s not that. You need to go and speak to your doctor and have a chat to her about palliative care”. (Carer 3) ‘The nurses were amazing. They were the go-to. . . It was them. There only in the daytime, of course. . .The nurses were really good at knowing what I needed to know, when’. (Carer 1) ‘We weren’t home three or four days before palliative care and the nursing system all called round to physically see us and give us information. . .And I really appreciated the way that all of them also judge our characters and didn’t hold anything back. . .she started talking to us, well, “I’m palliative care, you don’t really need that at the moment. I can see, but you’re dying, so you’re going to”. . .She didn’t pull any punches. She gathered very quickly from us this is the sort of people we are. And both of us really, really appreciated that. And it made us feel, well, I most certainly had no qualms with going ahead and ringing a nurse. So that was really good. I loved them dearly. And they were all so on the ball’. (Carer 7)

GP, General Practitioner.

### Theme 2: Knowledge, training and navigation

One of the most important things carers require is the practical knowledge they need to care for their loved ones. Often, carers do not feel they have the knowledge they need. Obtaining the knowledge required to carry out tasks to ensure quality care for the patient can be hampered by the lack of communication and sharing of knowledge from health professionals and also restricted by the day-to-day time constraints which are felt by carers. Therefore, recognizing the importance of knowledge sharing, training and navigation for carers is vital to support them in their role.

For most of our carers, their main concern was understanding the medications, dosage and side effects of these and how to undertake manual handling of the patient. For some of the carers, caring for the patient was further complicated by the patient having either dementia or Alzheimer’s. Essentially, carers would like a more personalized approach to helping them navigate the channels through which they can obtain resources, knowledge and training they need (Table 3).

**Table 3.** Knowledge, training and navigation.

Theme	Description	Exemplar quotes
Knowledge, training and navigation	Carers who were physically unable to undertake manual handling themselves were particularly vulnerable since they were strictly reliant on professional staff coming to the home. Most of the knowledge gained about these aspects of care came directly from home care nursing staff or from staff at the hospital:	‘Yeah, they said that when that time comes, he will be bedbound at some point. And yes, he’d need a nursing staff to come in to help him with sponge washes and turning him on bed, all that sort of thing, yes. I won’t be able to do any of that at all. Maybe a sponge bath, but not rolling him over and all that, no. Or lifting’. (Carer 15) ‘And my wife came with me, and the palliative nurse came, and she gave us advice, we learned how to change the bed. And lined up the medications, and what to use when. No, she had a gel. Yeah. Yeah. So, we were told how to do that’. (Carer 18) ‘The liver team through the specialists at [the hospital]. And he was through the palliative care at the [hospital]. Yes, I can ring palliative care and I can ring the liver team at [the hospital]. And I can ring RDNS. And I can also ring ECH. So, I’ve got plenty of contacts there’. (Carer 13)
	Some carers felt that the nurses provided such great advice that they were able to provide excellent care for the patient. On the other hand, others felt that even though they had the opportunity to engage with the nursing staff or care coordinators, they failed to consider the carer’s abilities:	‘And every time she would come, she would look at Mum’s medication, see how she is, look at her, and Mum didn’t have any bedsores. It’s because as soon as we saw one coming up, they told us how to treat it and we treated it straight away. And so, it never got to be a bedsore. . .’. (Carer 3) ‘But there is out of night times and weekends where it’s myself and my stepdad and that’s very much not reflected in the reports because these professionals come out for an hour and say, can you hold this? Can you do this, can you, whatever? And then decisions get made based on that and it’s not taken into account that. . . Or they’ll say that, oh, okay, you need two professionally trained people to transfer you, but we won’t provide a hoist or anything else. When I’m doing it, I’m fully untrained, young, smaller than my mum, transferring her by myself. There’s no manual handling training’. (Carer 2)
	Medication dosage, side effects and the purpose for prescribing certain drugs were also an area of concern. Some carers had clear instructions which put their mind at ease, others found solutions for their concerns and some felt not enough information was provided about the drugs. One had remorse regarding the strength of the drugs:	‘Yeah. There’s like the schedule of the medicine was posted in her house in the cupboard. And also, we have a schedule. Because I told you, they hired the carers. So, we have, who was in duty, in the morning, they will say, what did she do and what did you take. And then what do I have to do. . .’. (Carer 10) ‘My father disagreed, didn’t want to have a Webster-Pack and several times had made mistakes with taking incorrect things. And my mother, when she needed to have a lot of medications, she accepted that straight away and that was so easy. But again, some of those people aren’t even aware that you can get that from your pharmacist. . .And that’s been great, really happy to have had that because then you don’t have to worry about that. It’s one less thing that you need to be concerned about. And it’s pretty simple’. (Carer 11) ‘And it was very mechanical. It was very, “This is the dose, this is what you have. Oh, well, maybe we’ll cut this back a bit.” There was no explanation about what the drugs were. . . . We never got, “This is for your nausea. This is for. . . ” And he would set it that you took the. . . Oh, what hell’s the drug? I should remember. It’s a steroid for nausea. You’d take a tablet before you started the chemo, and then when you left you would have it that night. And then the next day. It used to hype him up. And they never explained what that was. Well, apparently, you’re supposed to gradually come off it the following Monday. Well, he just used to say to my husband, “Take for two days and then stop.” And that’s not good’. (Carer 16)
	The following carer expressed remorse during the interview that he may not have made the most of being with his father because he was not aware that the drugs were going to make him unconscious:	‘Yeah. And I didn’t know whether if it does knock him out, whether I can wake him up, or whether I could have time with him. All I did was sat in a room, and he couldn’t respond. So, I just talked to him, and played music. So, there were things I didn’t know, but I didn’t ask, I guess. You know?’. (Carer 18)
	Carers who had loved ones with special needs, such as dementia, the stress of caring was made much more complicated. One carer accessed the internet to gain information about the condition and what to expect as the patient deteriorates. Another claimed that there was a helpline that she could access, but there was a waiting list:	‘Well, I learned a lot about dementia, YouTube again, and anything I could get my hands on. So, I worked out how it works, what part of the brain is damaged and how that refers to what they’re expressing’. (Carer 6) ‘I’ve also had recent dealings with behaviour issues because my husband has also got some dementia, he’s also got seizures, which come on as well. And when those happen or when he’s unwell, he gets very irritable. So that’s difficult. I need to learn how to manage that. And so put me on a waiting list to get a dementia mentor that I can ring any time when I get into these sorts of situations so they can talk me through it’. (Carer 15)
	Overall, the ability to navigate the systems in order to find the right knowledge and training needed to provide optimal care for their loved ones needs to be more personalized:	‘I think, it’s got to be personalised. I don’t know quite how you would do it, but there has to be actual people walking around, that can point you in the right direction. . .Now, if there’d been someone, even at that stage, who said, “My name’s Mary or Bill, or whatever, and this is how it works.” We had no idea’. (Carer 16) ‘But, my push is for medical people, and also someone like me to navigate the system. Carers SA used to be at Morphett Vale face-to-face in a building. The worst thing they ever did was shut it down’. (Carer 5) ‘But there again, better on the phone rather than contact this website. I found the whole website being very impersonal. And, I do find I can’t be the only one that finds the whole concept of going online and filling out your details very cold and clinical. And also, am I doing the right thing?’. (Carer 7)

### Knowledge of and access to information and services: Organizing self-care support

We sought to determine how well-informed our carers were in knowing how to access support for themselves, either physically or emotionally. Findings suggest the barriers to gaining access to assistance rested on carer’s lack of knowledge, frustration with bureaucratic processes and lack of recognition from health care workers regarding their role. This is important, as the perceptions of and consideration for what carers do is the responsibility of those guiding and supporting them; government and health workers. This has a direct correlation with how carers see themselves and impacts how, when and what services are accessed or offered. Henceforth, carer self-identification does not exist in a vacuum but is shaped by how they are recognized within the current health services model.

### Theme 3: Carer recognition

In terms of carer recognition and identification, our findings suggest that carers may be recognized as such by various bureaucracies, health care workers and government organizations, but they also have to self-identify as a carer to seek help. Carers faced various difficulties in seeking government support due to the parameters of who qualifies as a carer. In our findings, carers’ understandings of what were available to them showed a lack of coordination and consistency (Table 4).

**Table 4.** Carer recognition.

Theme	Description	Exemplar quotes
Carer recognition	Carers who did know about government benefits or carers gateways did not really have a lot of success utilizing them and some, even when they had knowledge of them, chose not to register or apply for benefits. Some expressed frustration at both the limitations of carer recognition, and what it took to get assistance:	‘I get a part carer’s allowance. I can’t get the full carer’s one because I work in film industry, but not so much lately. . .. We had to get, to fill in all the forms, to get carer’s allowance when I was investigating this. The doctors that they sent him to, they say, “Can he feed himself. . . Can he use a knife and fork?” “Yes. Yes.” “Can he tie his shoelaces?” “Yes.” . . .The thing is, he would go out in 40 degree heat in his underwear walking down the street if I didn’t supervise. He would live on cocoa pops if I didn’t supervise. But technically, he can feed himself, he can shower, he can do those things. So, I was only entitled to a part allowance’. (Carer 8) ‘Not really, because my husband wasn’t physically. . . Because I always thought carer is helping someone on the toilet, helping them in the shower. I thought it was more like that. Someone said, “Oh, you should check if you can get any Centrelink benefits.” But to get Centrelink benefits, it does need to be more of that real physical help. It says, “It’s not enough if you’re making their meals, helping them with their meds, driving them to appointments, that’s not caring.” Okay. . .Well, I’m not a carer then. So, I didn’t even bother pursuing a government payment as I thought, “It’s not worth the trouble and I won’t get it anyway”’. (Carer 9)
	Carers felt that the application process was too difficult to facilitate:	‘Oh, I didn’t take part of [carers support organisation] as soon as I could have, because it was “get online and fill this all out,” which is understandable. It’s just like going to a new doctor’s, you’ve got to fill out your details. I understand that. . .I found it very difficult to do. . .the situation I was in, to take the time to sit in front of the computer because I didn’t want to be away from him for that long. . .’. (Carer 7) ‘Oh, with mum I needed the ID because she couldn’t come in because she’s pretty much housebound now. We needed ID for her to present to Centrelink or whatever it’s called now. . .So, that was difficult because she doesn’t have a driver’s license so there was no photo ID that I could present. So, then I had to get, I don’t know if it was a marriage certificate or something like that, to prove that it was her. So it got too hard and I abandoned the whole thing’. (Carer 15)
	Recognition of carers is also important from those health care workers they are in contact with during the care of their loved one. Some carers felt they were never recognized and as such, and were not provided information and support that they required:	‘Yeah, she [support worker] doesn’t like my stepdad. So, she, I think, actively chooses not to, to be honest. And I guess because I live there too. I don’t know if I’m a by-product of that. But I have only very rarely spoken to her ever. She isn’t it, yeah, the meetings and all that always during the day when we’re not there. My auntie is in a bit more direct contact with her when needed. . . . so yeah, like we don’t get sent any reports, any summaries any. . . anything’. (Carer 2)
	One participant spent 2 years caring for her dying husband and felt extremely upset by the lack of communication and information forthcoming to her during that time with regard for being recognized as a carer. She had made complaints about the specialist’s lack of communication to her GP and went in to see the specialist for a meeting about the treatment that her family had received:	‘Look, he was just so shocked. So, I did see him. It was not long after, within two weeks afterwards. And, he was full of apologies. And it was Covid. It was hugs and, “I’m sorry. You’ve had a hell of a two years.” Yeah, but no, he never said, “Do you need any help?” or anything. Because everyone was going, “How strong you are!” He would think that I could handle it. But I had a few health issues afterwards, which is obviously from all the stress and strain’. (Carer 16)
	One carer explained that recognition needs to be made from the outset from those health officials responsible for helping carers in their role. Again, a more proactive stance is called for:	‘What do you do? Where do you go? Who do you turn to? Maybe, I don’t know where that reference, you get a reference to go and you get referred to [carers support organisation] for example. When does that happen? Who identifies at what stage a person needs a referral? I don’t know whether that happens in the hospital, your GP or palliative care. One of those professional people need to be able to say “I think that person is caring.” Identifies your role now as caring. We don’t see it. But, somebody who’s standing back and can look from the outside in says, “Okay, that person now is a carer. They need support.” Rather than us putting our hand up’. (Carer 17)

GP, General Practitioner.

### Theme 4: Carer identification

Carer recognition and identification are linked. Identifying as a carer is more likely when others recognize their role; but it is also affected by the propensity to focus only on the patient and a familial sense a duty. This existential reality of being a carer directly influences help-seeking behaviour. Our findings also show that carers’ knowledge of and access to official support services for themselves often came through word of mouth from people who had been carers and members of nursing staff. Therefore, as with the theme of recognition, carer identification is more likely when their role of carer is recognized and acknowledged by others (Table 5).

**Table 5.** Carer identification.

Theme	Description	Exemplar quotes
Carer identification	In terms of self-identification, carers often saw their role as simply family duty. Carer burdens and being immersed in caring for their loved one resulted in a blinkered perception of their role due to their sole focus on the patient:	‘What I found in my situation is, I didn’t see myself as a carer for a very, very long time. I was a daughter and I was just looking after mum. It didn’t hit me. I didn’t get carers allowance. I got started getting carers allowance in the last 10 years because people kept saying to me, but you’re caring for your mum. I’m going, no, no, I’m just looking after mum. That’s what daughters do’. (Speaker 6, Focus Group 2) ‘When can we define ourselves as becoming a carer? And I think it’s that, it’s really leaning into the full-on stages where it’s really intense. It starts out as a very gradual thing and you’re not even thinking you’re a carer at that stage, you’re just helping your partner, and all of a sudden, not all of a sudden, but gradually becomes more intense. And then, you’re just enveloped in this whole situation. And from an outside of that, say, you’re certainly a carer, you don’t recognize that yourself, because by then, you’re so involved in what you’re doing, you can’t think about these other aspects’. (Carer 17)
	Focus on patient needs at the exclusion of their own needs was common for carers and affected help-seeking behaviours. Indeed, the link between identification and seeking care for themselves is significant:	‘I wasn’t at the time, and one of the big things is that I’m very, very bad at asking for help. I am someone who does for others. I’m not someone that does for myself’. (Carer 7) ‘I didn’t have time, yeah, or even to think about how I was doing’. (Carer 18) ‘And, we’re always so focused on mum’s care and what mum needs and all of that kind of thing that yeah, you kind of forget to seek that out yourself’. (Carer 2)
	Fortunately for some of our participants, they found out about self-care resources through word of mouth and through nursing staff: There was clearly a link between the recognition by others of their needs as carers which encouraged them to seek the help they needed:	‘And she had got me to become a member. So, I became a member that way. But no one in all my time caring for my mum, no one approached me about it. You’re a carer, and all my appointments with doctors and specialists, no one ever told me. I didn’t even know about carer’s allowance’. (Carer 3) ‘One of the women there said to me, “Get onto Carer’s SA.” Because, she was looking after her mum. So, I think she told me about it and I went online, or I rang them up. That’s right, I rang them up and then they did an assessment on the phone’. (Carer 9) ‘The nurses and palliative care and everybody gave me information for being able to get carer’s help. It wasn’t until I got here that I actually asked for carer support, and I did use a couple of phone calls for mental health support, which was really good’. (Carer 7) ‘[Nurse] would ask me, we’d sit down and have a cup of coffee. And then it was all about “Okay girls, how are you doing?” So, it came from the [nurse], the IDN nurse. And, every time one came in, they always would ask, “How are you doing?” So, from them, we got it’. (Carer 3)
	Some participants did seek official counselling from a professional but this predominantly occurred post-bereavement. Carer’s 17 and 18, our two male participants, sought or were offered formal help and another mentioned how difficult it had been to organize an appropriate counsellor who specialized in grief and bereavement:	‘And once I made contact with them, and I’ve got a card, [bereavement support coordinator], she’s one of the counsellors, I’ve spoken with her and she’s just sent me a card the other day, inviting me to connect at any time in an ongoing way with counselling. Yeah. [She’s] good. . .And so she’s lovely. And I’ve been a counsellor as well. And just to hear [her], I was talking to [her] last night, and it’s just about being there for someone. . . .so they’ve got a good team here. That follow up is very important’. (Carer 17) ‘And I was until even a week after Dad passed away, I think it took a while for it to really hit me, and it’s just. . . I still do talk about him, when I talk to [name of counsellor], or I talk to my counsellor. . .that’s somebody that I already knew’. (Carer 18) ‘I think it was about a month after my husband died that I thought I should look at trying to find a psychologist with grief and bereavement skills and it’ll be, I’ve got my first appointment with her in about three weeks, so it’ll be nearly six months since my husband died for my first appointment. That’s how long it’s taken to find someone who’s local. If I’ve been willing to drive up to Adelaide, it might have been quicker, but I wasn’t willing to do that. But, that’s kind of typical of any, to get in with any psychologist or psychiatrist, it’s usually about a six month wait. And then there is a cost involved. Even I’ve got private health, I know there’s going to be a gap payment. And again, I’m in a situation I can afford that. But you know, you just know there’s a lot of people out there that’s not, but they can’t afford it. It should be, ideally everyone should be able to get a few sessions with a bereavement counsellor’. (Carer 9, Focus Group 1)
	Informal support groups seemed to be preferred by carers due to the value placed on the comfort and support felt from those who had been through a similar journey:	‘Carers SA was my main support. So, every month I would go to a Carers SA group for carers. I also went to Fleurieu Cancer Support Foundation. They have a craft group run by women who’ve both had cancer themselves or have lost someone to cancer. And I used to go to that most Fridays. It’s only a small group, but again, there was a couple women there who’d lost their husbands to cancer. So just having people who’d been through this already was a big help. Then my other main support was through church. My husband and I were both members at a local church’. (Carer 9) ‘Likewise, you just need to speak to people who have been down the path or people. Other people want to be helpful, but the people who have experienced it get it. And they also get it that you’ve not got a partner’. (Carer 16) ‘I’m a part of a support group here in Murray Bridge that it’s a support group for people who’ve lost someone to suicide. I haven’t lost anybody, but I facilitate that group. . .I think about grief and we talk about the stages of grief and how that works. . .When you’re in that space with this group of people, words aren’t necessary. They sit and they know what the other person’s been through. . .I think that’s where we are talking about that understanding of our experience as carers, that you really need to go through it before you can get a handle on it. Grief as well, grief is a personal journey that no counsellor can stand up and tell you, There are four stages and this is what you can expect. Then you go into this stage. It doesn’t, does it?’. (Carer 17)

## Discussion

Our research shows that carers’ access to information, knowledge and support needs in caring for the patient and themselves continue to be compromised. The widely acknowledged contribution they make to health care and the formal recognition of their importance through formal channels do not reflect the lived experience of our carers. This is a paradox, considering that our findings suggest that carer recognition and self-identification are central to where, what and how information is sourced and whether or not they experience adequate support.

Our findings show that with regard to accessing information and knowledge to assist in patient care, there is inadequate and inconsistent communication between General Practitioners and carers regarding transitioning to palliative care and the provision of resources and knowledge for patients in this category. A couple of families had good dialogue with their GP regarding palliative care and felt more prepared for their role. Many family caregivers rely on their doctors to provide accurate prognoses along with information regarding hospice care.^38^ GP’s are in an excellent position to initiate conversations regarding the role and benefit of palliative support services early in the disease trajectory.^39^ However, as is evident from some of our carers, GP’s do not take full advantage of their potential in this area and this lack of open dialogue is viewed as a missed opportunity by carers to obtain knowledge, resources and support required for patient care. Our research concurs with another study which found that low satisfaction with GP’s is a strong predictor of the lack of services involved in patient care whereas positive support from nurses is a strong predictor of the patient receiving multiple avenues of support with hospice care.^15^ Some of our carers, as also reflected in other studies, agreed that even if hospice is discussed, it is too late and often without provision of information, resources or referral.^38,40^

GPs often have a reactive stance to communication, only responding to carers’ needs when asked.^13^ This is echoed by one of our participants, who said, ‘*Only if you ask: if you don’t ask, you don’t get anything*’. A lack of proactive guidance with regard to information and support for carers of palliative patients can lead to a number of issues. Some suggest that a lack of GP referrals to specialist palliative care can be due to their management of the symptoms and a lack of vocational training in palliative care more broadly.^41^ This implies that if they feel they are managing the patient, then palliative care may be delayed thus resulting in a lack of discussion with carers. Even though our findings do not conclusively suggest this, what is certain is that there seems to be a lack of coordinated focus on the importance of discussing palliative care. This has the potential to negatively impact the quality of patient care and the ability of our carers to cope with their role. Since carers need a good level of trust and often don’t ask for information from professionals unless invited to do so,^12^ GP’s need to take a more proactive approach to discussing palliative care information and resources with carers.

With respect to nurses as potential gateways for knowledge and resources for palliative care, the reaction from our participants was largely positive. Many of our participants received information they needed from nursing staff visiting the home. However, the caveat here is that any knowledge or information imparted to carers at this stage is often after palliative care referrals have already been organized. Henceforth, although our participants were grateful to the nurses for sharing their knowledge, this information and knowledge regarding resources for patient care came much later in the disease trajectory. GPs, nurses and other health professionals involved in patient care should be mindful of ensuring knowledge and resources regarding palliative care are imparted to the carer as early in the disease trajectory as possible. Furthermore, attention needs to be focused on the important part that carers play and the issues they face and how this impacts patient outcomes more broadly. As is indicated from other studies, open communication, family meetings and the sharing of care goals leads to significant positive outcomes for both patient and carer.^42,43^ Hence, this is one way that early interactions between carers and health professionals can seek to reduce the deficits in this area.

The unmet needs of carers with regard to knowledge, resources and information required to carry out their role is well documented.^17,44^ Our theme of knowledge, training and navigation revealed two main findings. The first is that carers were very appreciative to receive hands-on advice with regard to patient care such as manual handling and guidance and advice on resources. The second were those who did not receive this advice sought to navigate online to find information they needed, particularly when it came to medications and their side effects. Indeed, much of what the carers experienced in terms of learning and training largely depended on positive engagement with palliative care staff doing home visits and hospital staff being open to receiving phone calls from concerned carers. Carers who had opportunities to learn about lifting and bed sores, for example, were very appreciative and seemed to provide them with piece of mind. This is because carers who are more informed and have received some education and instruction experience a feeling of preparedness and confidence in their tasks.^11,45^ Preparedness is not only essential for carers to sustain their role but leads to reduced caregiver strain.^42^

The second finding was that those who did not receive the appropriate training, advice and resources required to carry out patient care were left to figure this out on their own through the internet or helplines. Particularly vulnerable were those carers looking after patients with either dementia or Alzheimer’s and those who did not receive home help. Our findings show that these groups are not receiving the information they need and are experiencing frustration caused by this lack of knowledge. Of particular note were issues regarding medications, symptom control and side effects, which can be a source of concern when patient behaviours are unpredictable and difficult to manage. Finally, carers not receiving home visits from palliative care staff are at particular risk of being left without the knowledge, training and resources they need since there is little exposure to health teams and opportunities for dialogue. Fundamentally, the overwhelming response from our carers regarding this theme was that they prefer a personalized approach, either face-to-face or by telephone, to help them navigate terrains to find the information and resources they need for patient care.

Our theme of carer recognition and identification under the category of self-care was particularly interesting. Considering carers face specific physical and mental stresses such as physical exhaustion, emotional distress, social isolation and financial burdens and face great risks to their mental health,^20,21^ one would assume that the importance of recognizing carers by health professionals would be paramount. As such, self-care avenues of support should be made particularly blatant to carers. Indeed, the recognition of their vulnerability and importance to the health system is reflected by the World Health Organization who claim that health providers should be ‘approaching patients and their caregivers as a “unit of care,” focusing on the overall well-being of the patient-caregiver dyad rather than just on the patient’.^46^ However, whatever official recognition carers are afforded; this was not the predominant experience of our carers. Recognizing carers’ needs and burdens and offering help and self-care information was sporadic and reactive rather than proactive on the part of health teams. Nurses fared much better in this category than GP’s, but carer recognition was still severely lacking.

Studies show that help-seeking behaviours of overburdened carers are low unless there is a proactive stance by health professionals to offer help.^11,13^ Our study supports this evidence. Carers are already reluctant to recognize the important role they are playing and are unlikely to reach out for emotional or financial help. Carers in our study found out about self-help avenues mainly through carers who had been through a similar journey, or home care nurses. Again, where there was some kind of face-to-face dialogue, carers were more likely to consider seeking out help. However, what is evident from this cohort is that the gap between the official narrative of carer contribution and recognition and the lived experience of carers is still too wide. Therefore, the negative effects of the lack of carer recognition from this research cannot be overstated.

A particular novel finding of our research, which the authors have not encountered elsewhere, was the intrinsic links between carer recognition and carer identification which impact help-seeking behaviours. First, our evidence suggests that help-seeking behaviours of carers are firmly grounded in self-perception. This was a significant finding as it had direct correlation with receiving support. Self-care is vital to seeking help, and identifying as a carer is the first step. However, carers in our study, as has been shown elsewhere, are often strongly influenced by internal senses of duty and love and also social and cultural expectations of caring without the expectation of recognition.^8^ Many felt they were just carrying out their responsibility and duty to their family and others sustained a caring role for a considerable time before they identified as a carer. Obviously, this puts carers at risk of not seeking the support they require and makes them vulnerable to developing their own health issues. Indeed, even where carers were recognized and identified as carers by health teams, such as home care nurses, having that recognition was clearly appreciated, whether or not the carer took the next step in self-care. What this finding suggests is that health professionals should be responsible to identify the carers in our community and in so doing, be sharing knowledge about support and information resources with them. This is a simple policy but, as our evidence reveals, a dialogue that is absent or lacking. Indeed, it is the carers that prefer to receive guidance face-to-face, so this is a blatant missed opportunity for our health professionals to support carers.

The existence of government initiatives, such as the Carers’ SA online portal, was widely unknown to the carers in our cohort. With the exception of one carer (9), this site was rarely utilized. Hence, even though one could argue that success of other web initiatives seems to rest on being co-designed by carers,^25,27^ our study suggests that this would not make a difference to its utilization. Part of the reason for this lay with the carer themselves. Most carers can search the internet for information, but not in a systematic way. Carers are time-poor, stressed, patient-focused and often do not self-identify. Indeed, the barriers created by a lack of self-identification cannot be overstated. Carers who do not identify as carers do not seek avenues for self-care. This is particularly detrimental for a group highly susceptible to extreme mental, emotional and financial burdens.^20,21^ Indeed, financial assistance was also under-accessed for the same reason. Many carers found that government definitions of carers are too narrow and the online application process too taxing. Carers are time-poor, and many felt that the financial assistance was not worth the time and processes required to apply.

Just as lack of self-identification affects help-seeking behaviour, so does patient focus. We found that carers did not seek out professional counselling whilst they were undertaking their caring role but were more inclined to do so post-bereavement. This supports the evidence suggesting that psychosocial and physical burdens are acute not just during the caring process but during this phase.^22^ It also suggests that the removal of focus on patient care, family obligations and responsibility, the socio-cultural elements of caring permits carers to again consider their own needs. This further attests to the significance of carer self-identification. Carers loose themselves in patient-focused objectives. Once this focus is removed, they again return to thinking about their own needs and self-care.

Another finding with regard to self-care and post-bereavement was a general lack of coordinated approach by health teams in following up with carers. However, many carers spoke about and engaged with more informal forms of support such as family, friends and even some community groups. It was mentioned time and again that carers gain comfort and support by engaging with those who have been through a similar experience. Carers from our study prefer not only to speak with professionals who are trained in the specifics of bereavement, but also to be around and supported by those in the community who have been carers and experienced loss in the same context. This shared understanding creates a trusted mode of support where people can lean on each other. Even those who did not officially seek out self-care options at the time of caring had a propensity to help others post-bereavement by sharing their knowledge and support with others going through a similar experience.

The authors believe that these findings provide a strong indicator that a ‘compassionate communities’ approach to sharing information, knowledge and support for carers of palliative patients would be very effective. In this public health approach, community development and community capacity building serve to enhance knowledge of end-of-life care, where carers can make more informed decisions and utilize support systems more effectively.^47^ Furthermore, it recognizes the limits of health service provision and focuses more on bolstering a social capital approach to care.^48^ Indeed, our findings show that grassroots community support groups for carers run by those with first-hand experience would be more likely to be utilized and benefit the wider carer community than more official, formal channels like websites.

## Strengths and limitations

Recruitment for this research proved more challenging and prolonged than expected. We believe this, in part, relates to the particular stresses inherent to this particular group, the part that bereavement plays in recovery, and our attempt to garner experiences from those currently undertaking a caring role. However, although recruitment was slow, we had enough interest from participants to conduct a total of three focus groups. This gave us a valuable opportunity for carers to provide feedback on some of our preliminary findings and share and discuss issues and experiences as these findings pertained to them, essentially allowing for triangulation. Further, it provided an opportunity for carers to provide recommendations on how to move forward to overcome some of the challenges that carers face. Finally, we were fortunate to recruit participants from different socio-economic and cultural backgrounds. Even though none of these participants required interpreters, they still provided more diverse perspectives on end-of-life care as it pertained to their belief systems, cultural traditions and religion.

We recognize the limitations present based on the number of participants willing to take part and the lack of response we received from those from non-English speaking backgrounds. Attempts were made to reach out to Culturally and Linguistically Diverse (CALD) community organizations to advertise our research but unfortunately received no responses within our working time frame. We recognize how important this data would be in understanding the additional challenges these particular groups would face is accessing knowledge, services and support as unpaid carers.

## Implications for policy and future research

The gap between the formal recognition of the importance of unpaid carers and the reality of the lived experience of this group reveals various implications for future health policy. First and foremost, information regarding resources and knowledge of support is sporadic and lacks focused coordination. A concerted response to this should be early identification of carers in our community by health teams, along with a proactive engagement with them in order to provide the knowledge, support and resources they require. Further, our findings regarding a lack of engagement from GPs is particularly concerning, considering they are the first to coordinate the needs of patients and should be the first to identify and help carers to carry out their role and ensure their well-being is also prioritized. Furthermore, since we found that help-seeking behaviours are intrinsically linked to self-identification and recognition, it is the responsibility of health professionals to identify, recognize and open dialogue with carers. This would not only help prevent the emotional and physical burdens they face as individuals but provide personalized one-to-one discussions and support that, from this research, proves to be not only preferred but more likely to be effective.

Government financial assistant schemes for carers need to be less confusing, convoluted and time consuming. Carers are time-poor, and some have difficulty when all applications for resources are strictly online. This again attests to the personalized approaches that carers need and suggestions from them that a ‘care navigator’ helper, someone they could personally visit for help, should be in existence. Furthermore, government application processes need to make allowances for and consider those older carers who have difficulties with technology and often rely on others to help them navigate these systems. They must also recognize that where carers fail to receive this personalized help, applying for such assistance is often abandoned.

Future research should incorporate, where possible, participants from CALD communities and carers of palliative care patients suffering from either dementia or Alzheimer’s. These two groups should be recognized as particularly vulnerable since they face significantly more complex challenges in caring for patients in addition to those already identified in this research.

## Conclusion

Carers of patients requiring palliative care need to be recognized for the vital role they play in palliative care provision more broadly. They are integral to positive outcomes for this patient group and will be increasingly more important in the future by relieving considerable strains on health systems which are predicted to rise. The gap between the formal recognition of their importance and the reality of their lived experience is clearly evident from our findings. Carers continue to feel overburdened and ill equipped to carry out their role not due to lack of services, but due to a lack of knowledge, information and coordination of these services and hope to obtain what they need in this area with a more personalized approach. Furthermore, help-seeking behaviours of carers are intrinsically linked to self-identification and recognition. Since patient focus is paramount for carers, this identification and recognition needs to be initiated in a proactive instead of reactive manner from health professionals in the community. Positive patient outcomes and carer well-being requires an open dialogue and a shift to a more focused, intentional effort by coordinated health teams to ensure carers are prepared for their role and are emotionally supported to sustain it.

## Supplemental Material

sj-docx-1-pcr-10.1177_26323524241255386 – Supplemental material for Voices of unpaid carers: problems and prospects in accessing palliative care and self-care information, resources and services Supplemental material, sj-docx-1-pcr-10.1177_26323524241255386 for Voices of unpaid carers: problems and prospects in accessing palliative care and self-care information, resources and services by Kristine Van Dinther and Sara Javanparast in Palliative Care and Social Practice

## Declarations

**Ethics approval and consent to participate:** Ethics approval was granted by the Flinders University Human Research Ethics Committee (project ID 4559). Study participants provided their written consent to participate.

**Consent for publication:** Not applicable.

**Availability of data and materials:** Conditional upon contact with corresponding author.
